# Supplementary material for: The Torreya grandis genome illuminates the origin and evolution of gymnosperm-specific sciadonic acid biosynthesis
Source: Nat Commun. 2023 Mar 10;14:1315. doi: 10.1038/s41467-023-37038-2 (PMC10006428; doi:10.1038/s41467-023-37038-2)
Supplement: Supplementary file 1 — Supplementary Information [file 41467_2023_37038_MOESM1_ESM.pdf]

The *Torreya grandis* genome illuminates the origin and evolution of gymnosperm-specific sciadonic acid biosynthesis

Lou *et al.*

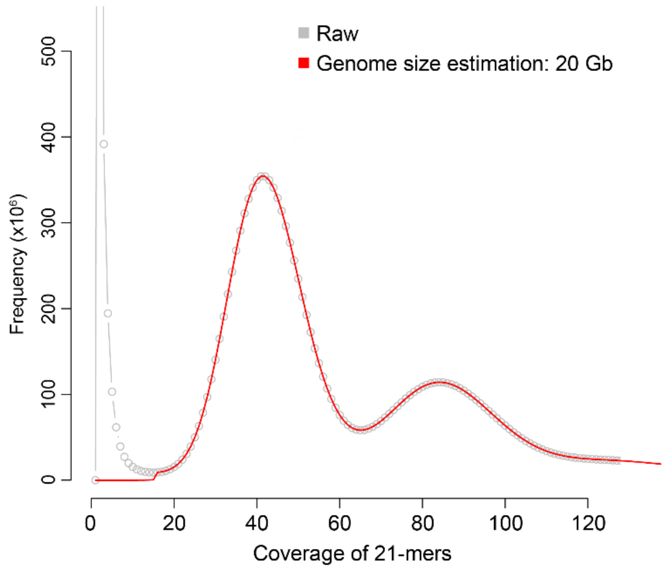

Supplementary Figure 1. 21-mer spectrum of the *T. grandis* Illumina reads. Genome size was estimated using the FindGSE program (https://github.com/tiramisutes/findGSE).

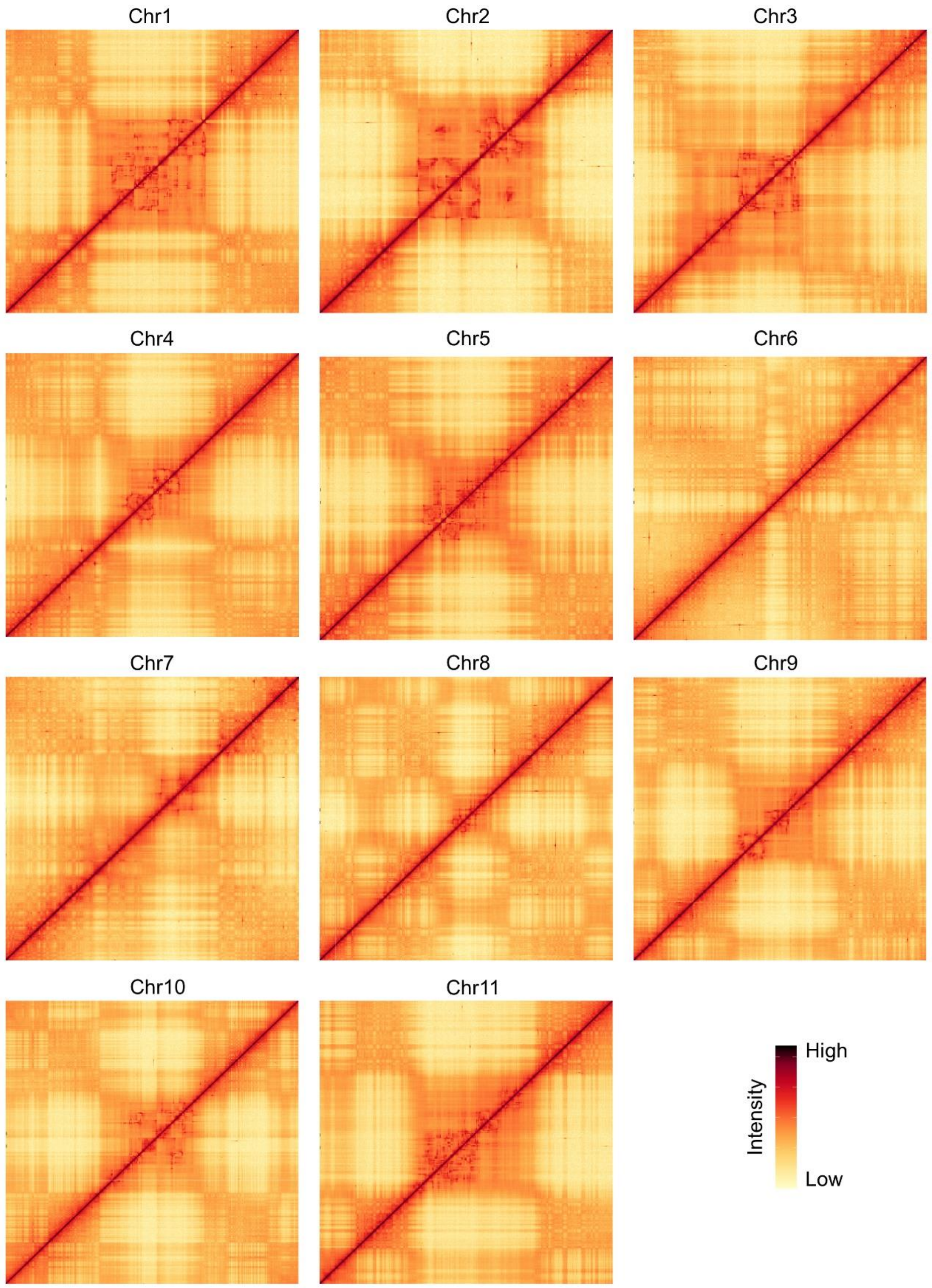

Supplementary Figure 2. Hi-C interaction map of each chromosome of the *T. grandis* assembly.

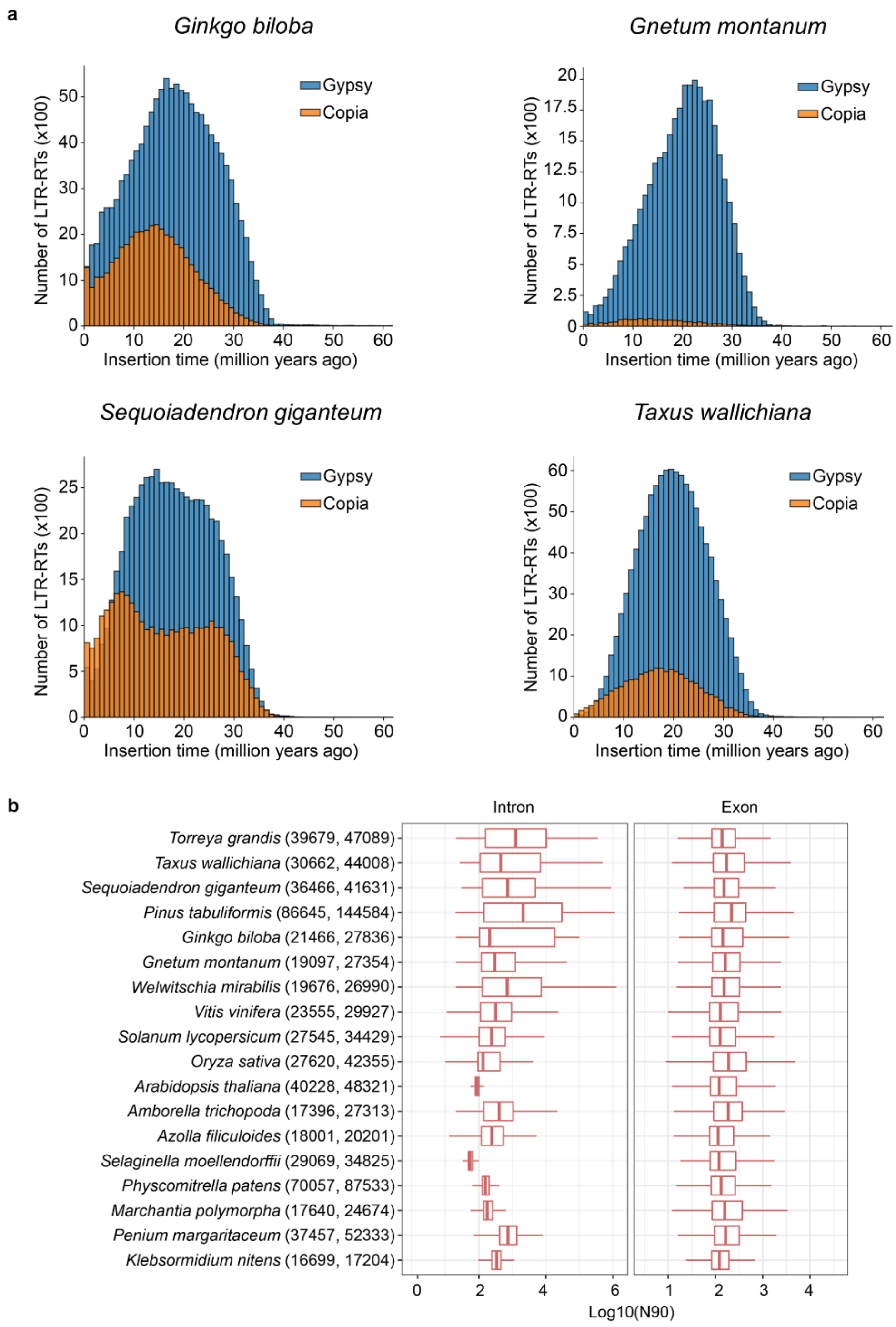

Supplementary Figure 3. Distribution of LTR-RTs in selected genomes of gymnosperms. (**a**) Expansion of *Copia* and *Gypsy* families in four selected genomes. (**b**) Boxplot of intron/exon lengths in different plant species. The N90 size of intron/exon for each gene was calculated and used for plot. For each boxplot, the lower and upper bounds of the box indicate the first and third quartiles, respectively, and the center line indicates the median. Numbers following species name indicate the quantity of introns (first number) and exons (second number) used for boxplot.

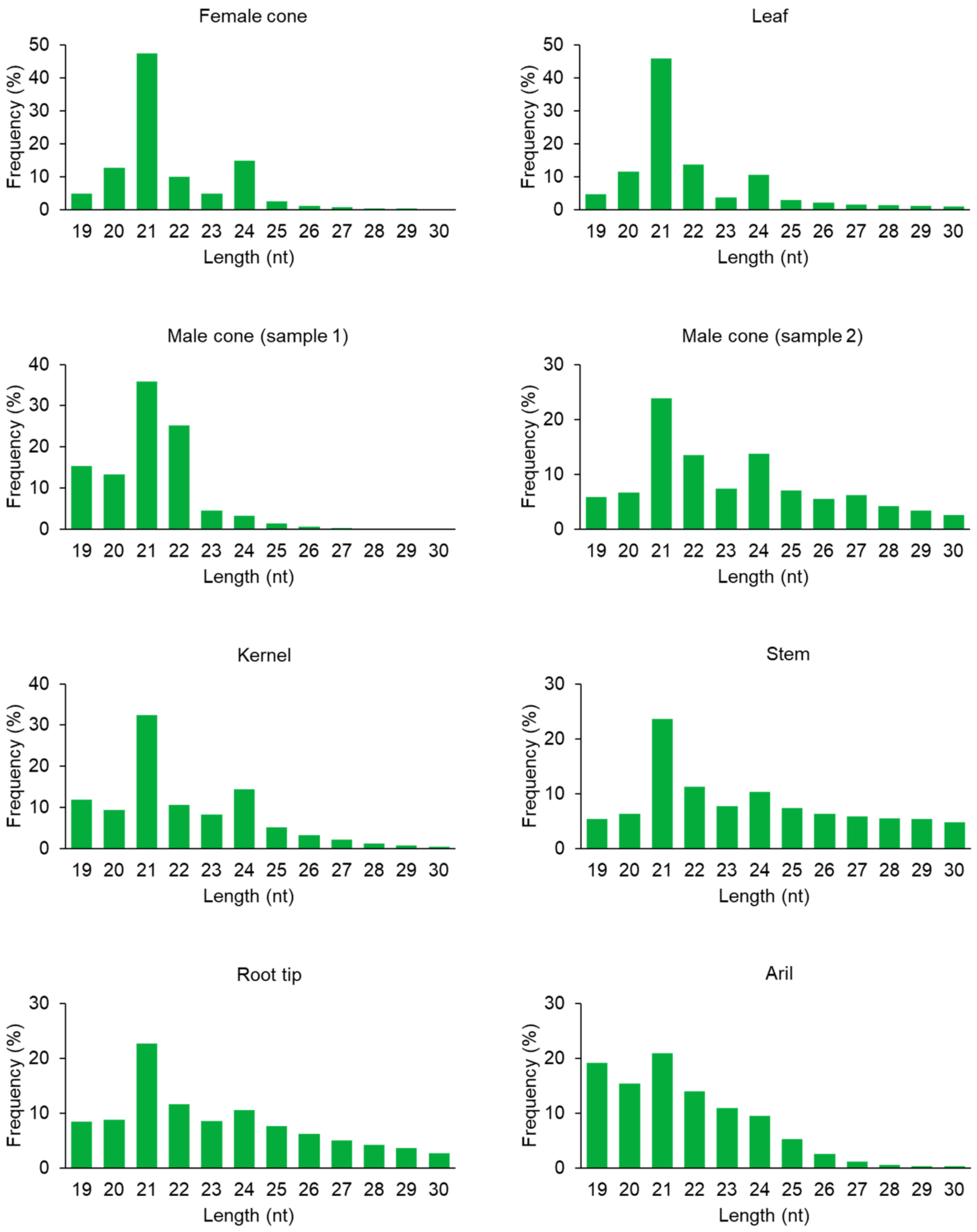

Supplementary Figure 4. Size distribution of sRNAs in different *T. grandis* tissues. Percentages of sRNAs with different lengths are shown.

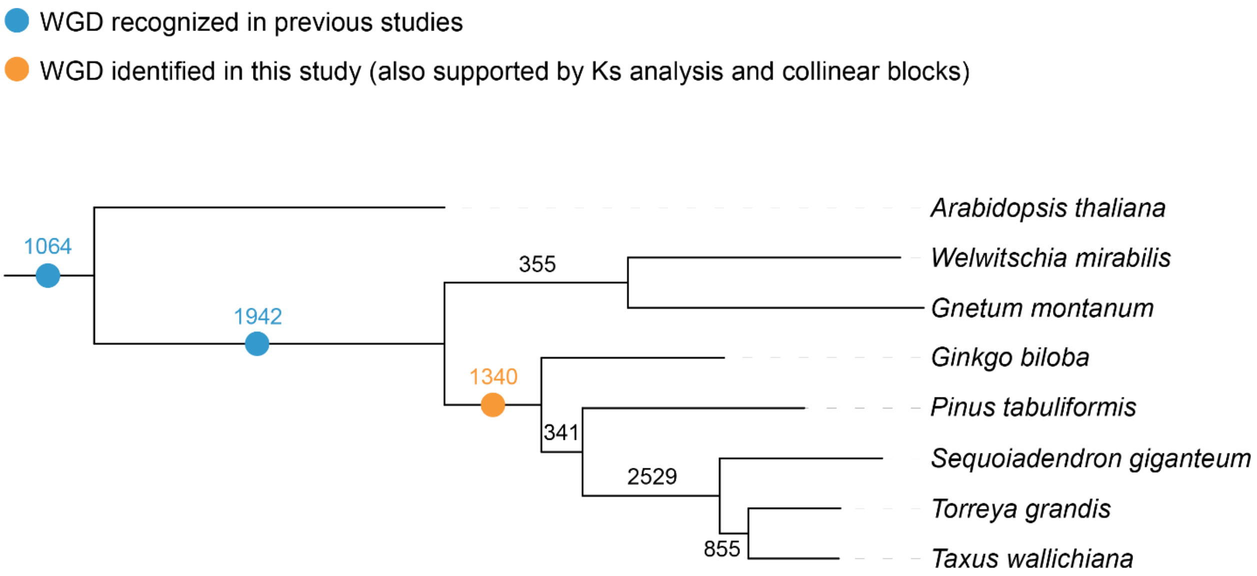

Supplementary Figure 5. Tree-based WGD inference. Numbers on branches represent the quantity of duplicated gene families based on reconciliation of gene trees and species trees. Branches with colored pies imply the presence of WGD.

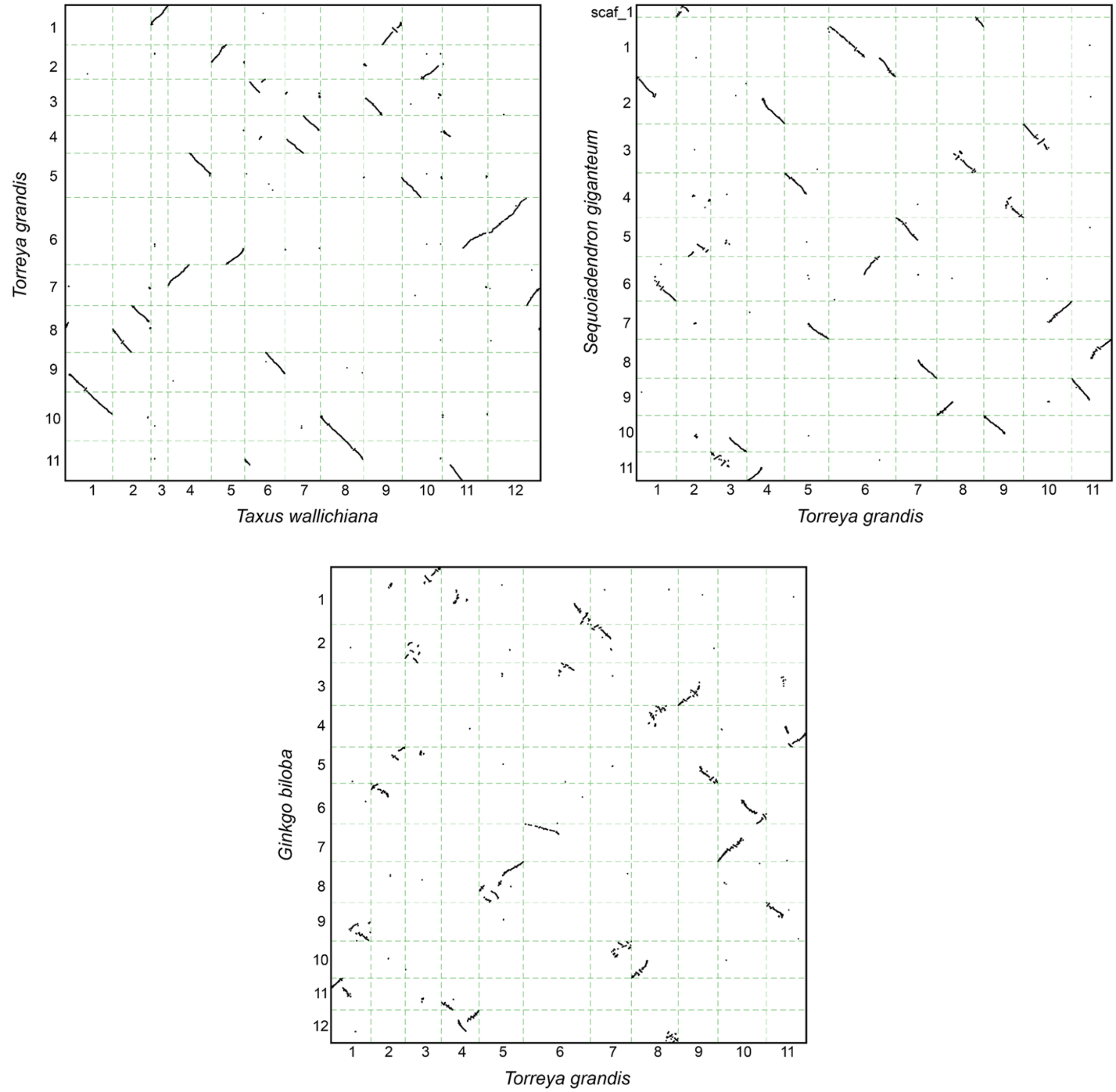

Supplementary Figure 6. Collinearity between genomes of *T. grandis, S. gigantenum* and *G. biloba*. Numbers on x- and y-axis indicate chromosomes. The degree of collinearity correlates with the phylogenetic distance of the species. The genome of *T. grandis* shows an overall higher level of collinearity with the *S. gigantenum* genome than with the *G. biloba* genome.

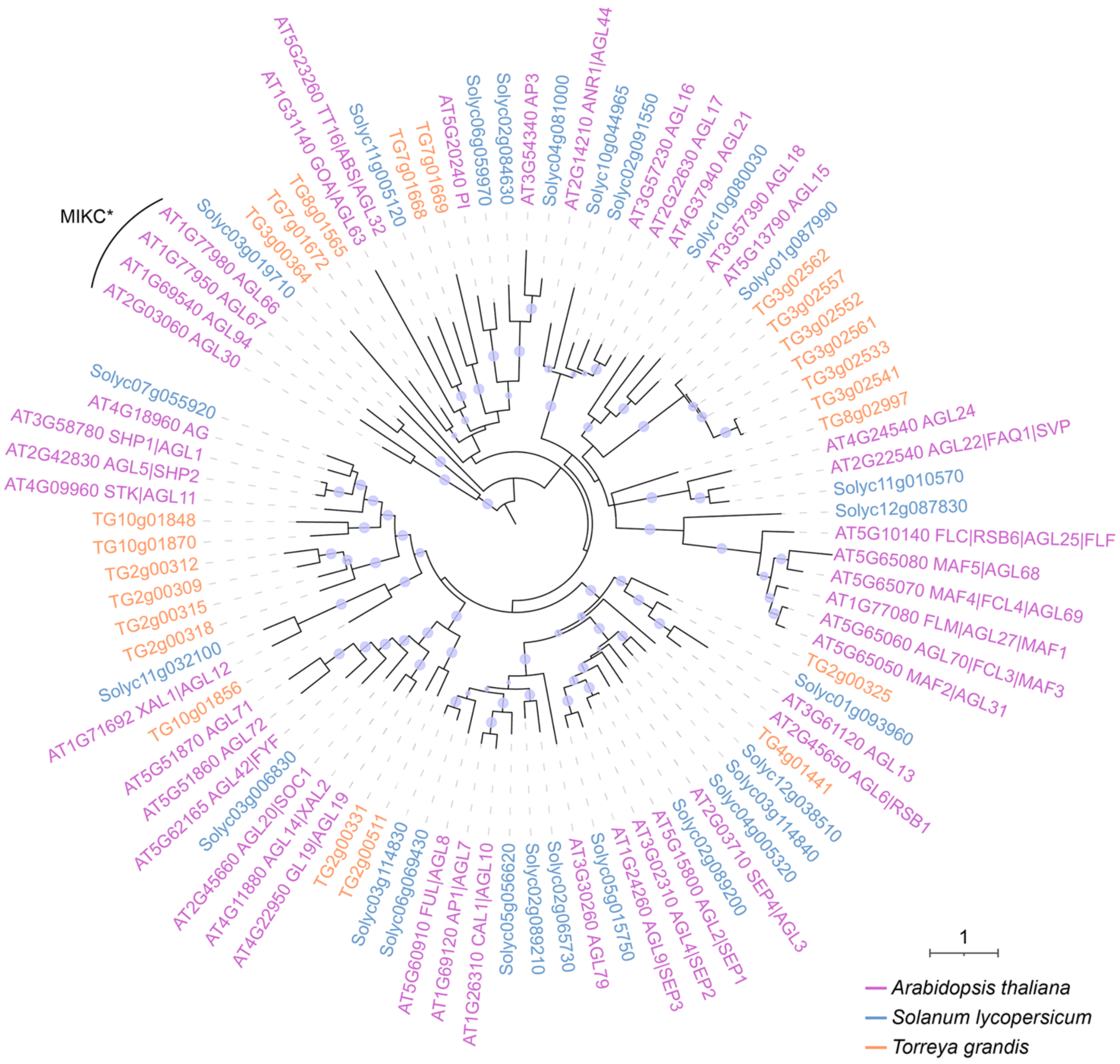

Supplementary Figure 7. Phylogeny of MIKC^{C} type MADS-box genes. Protein sequences of MADS-box family genes were aligned using MAFFT with the linsi mode, and the phylogenetic tree was constructed using IQ-Tree with the best-fitting model (JTT+F+I+G4) and 1000 bootstrap replicates. Branches with bootstrap support greater than 80% were labeled with light blue pies. The tree was rooted with MIKC* type MADS-box genes.

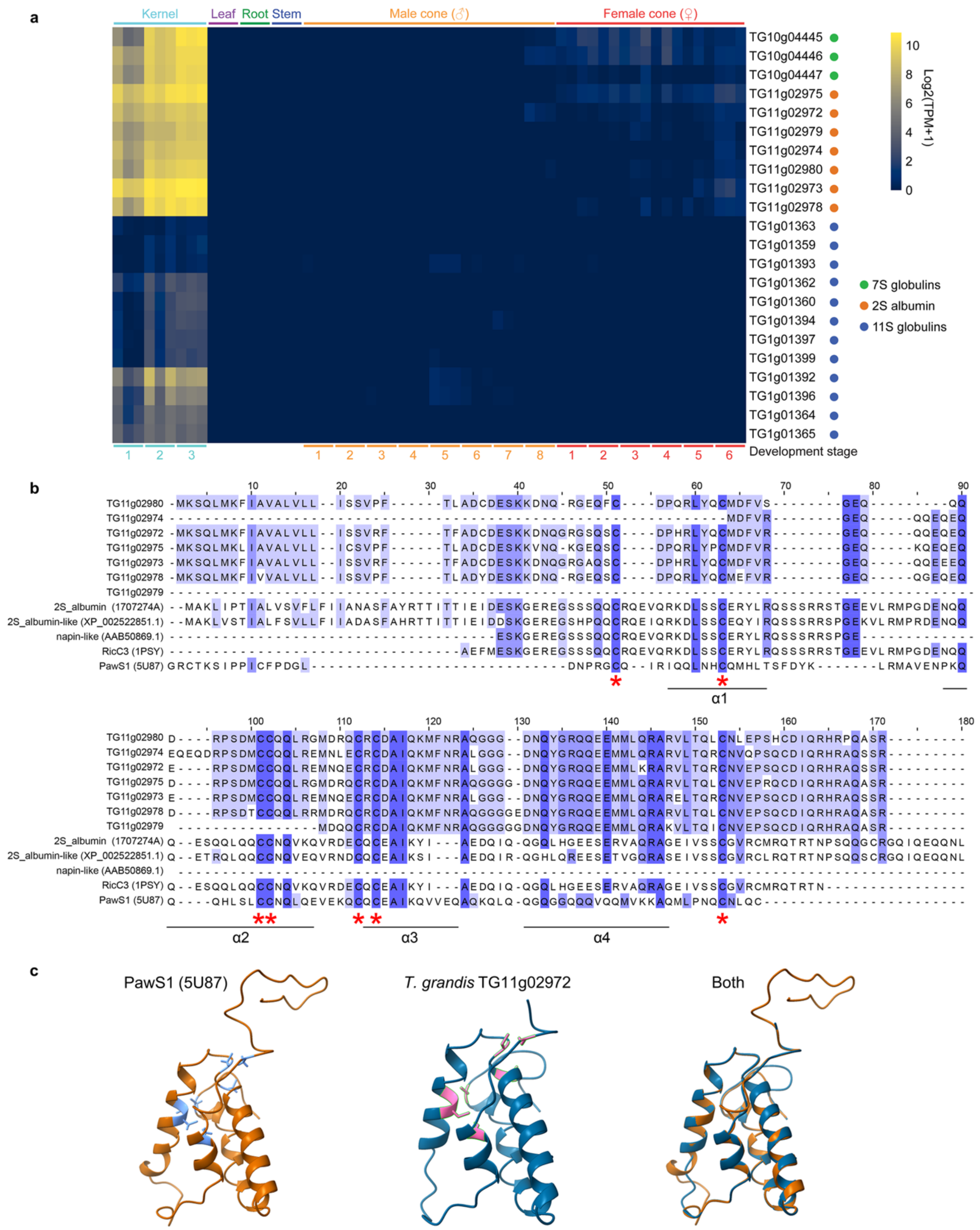

Supplementary Figure 8. Seed storage proteins in *T. grandis*. (**a**) Expression of genes encoding seed storage proteins in vegetative and reproductive organs of *T. grandis*. (**b**) Sequence characteristics of 2S albumin family proteins. Red asterisks indicate conserved cysteines. Protein sequences forming the α-helices are underlined. (**c**) Homology modeling of 2S albumin proteins from *T. grandis* and sunflower (PawS1).

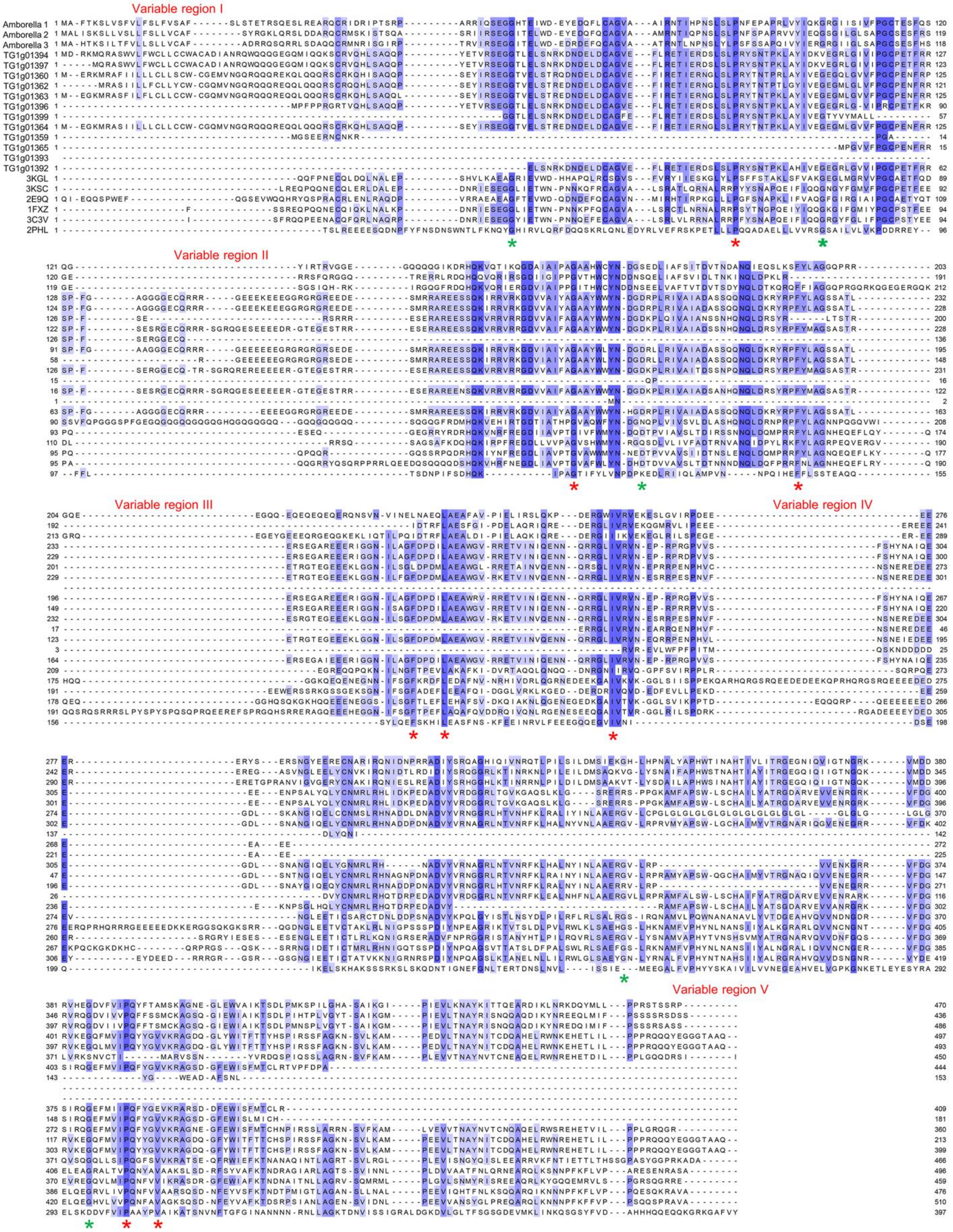

Supplementary Figure 9. Sequences characteristics of 7S globulins. Red and green asterisks show functionally important residues that are conserved and variable in *T. grandis*, respectively.

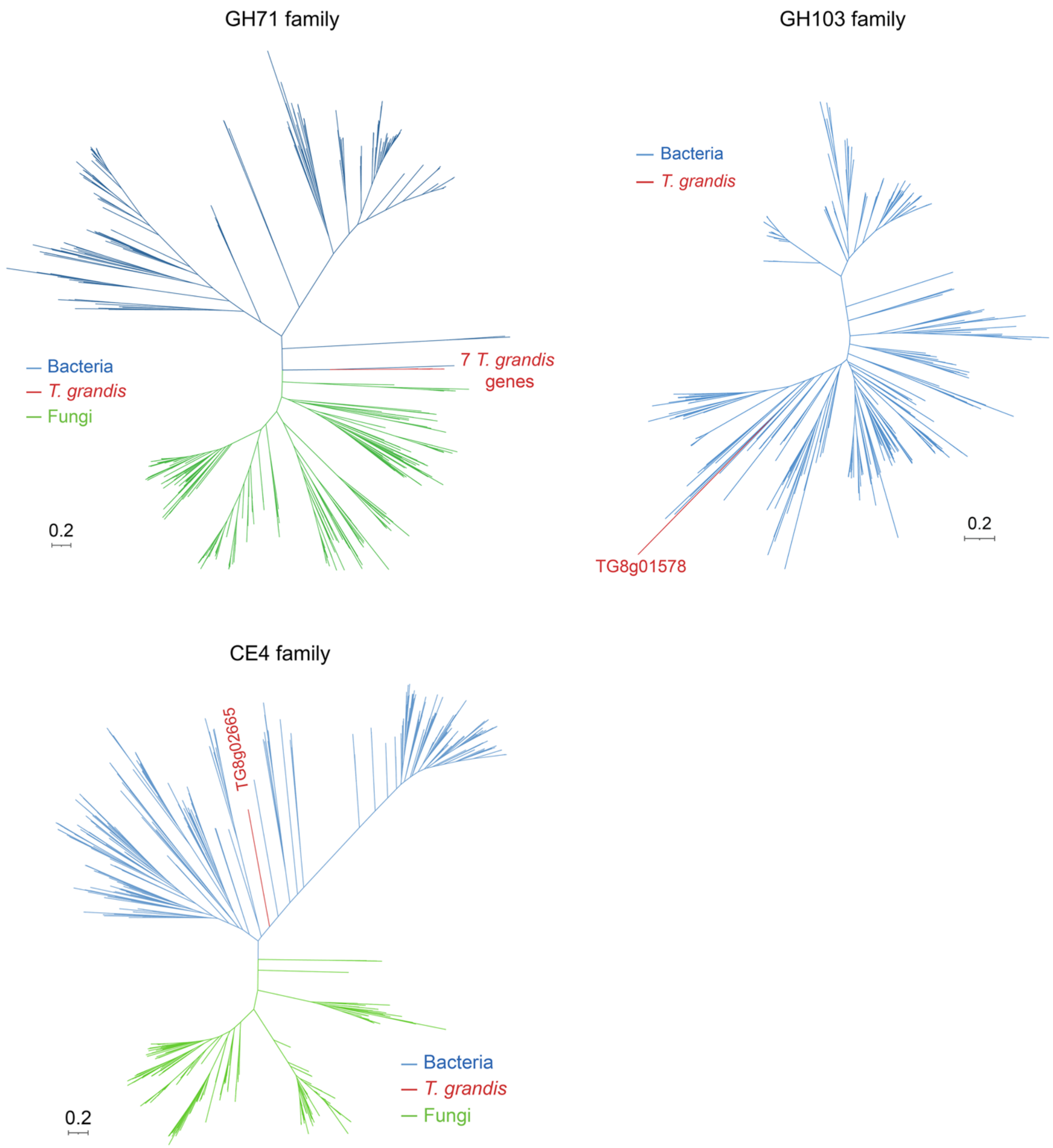

Supplementary Figure 10. Phylogeny of CAZyme genes. Protein sequences were aligned using MAFFT with the linsi mode, and the phylogenetic trees were constructed using IQ-Tree with the best-fitting models (Q.pfam+I+G4 for all three families) and 1000 bootstrap replicates.

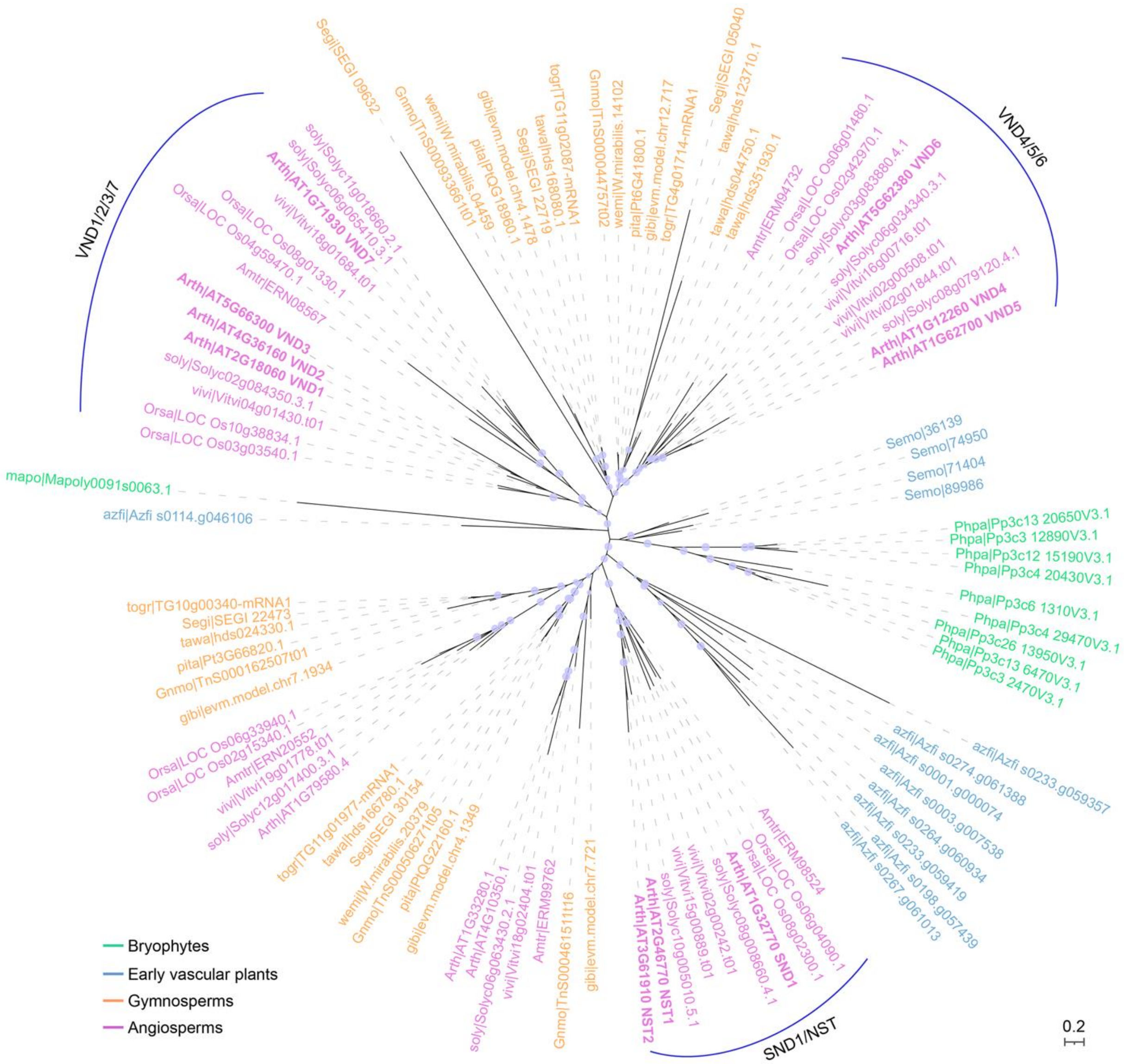

Supplementary Figure 11. Phylogeny of VND, NST and SND family genes. Protein sequences were aligned using MAFFT with the linsi mode, and the phylogenetic tree was constructed using IQ-Tree with the best-fitting model (JTT+I+G4) and 1000 bootstrap replicates. Branches with bootstrap support greater than 80% were labeled with light blue pies.

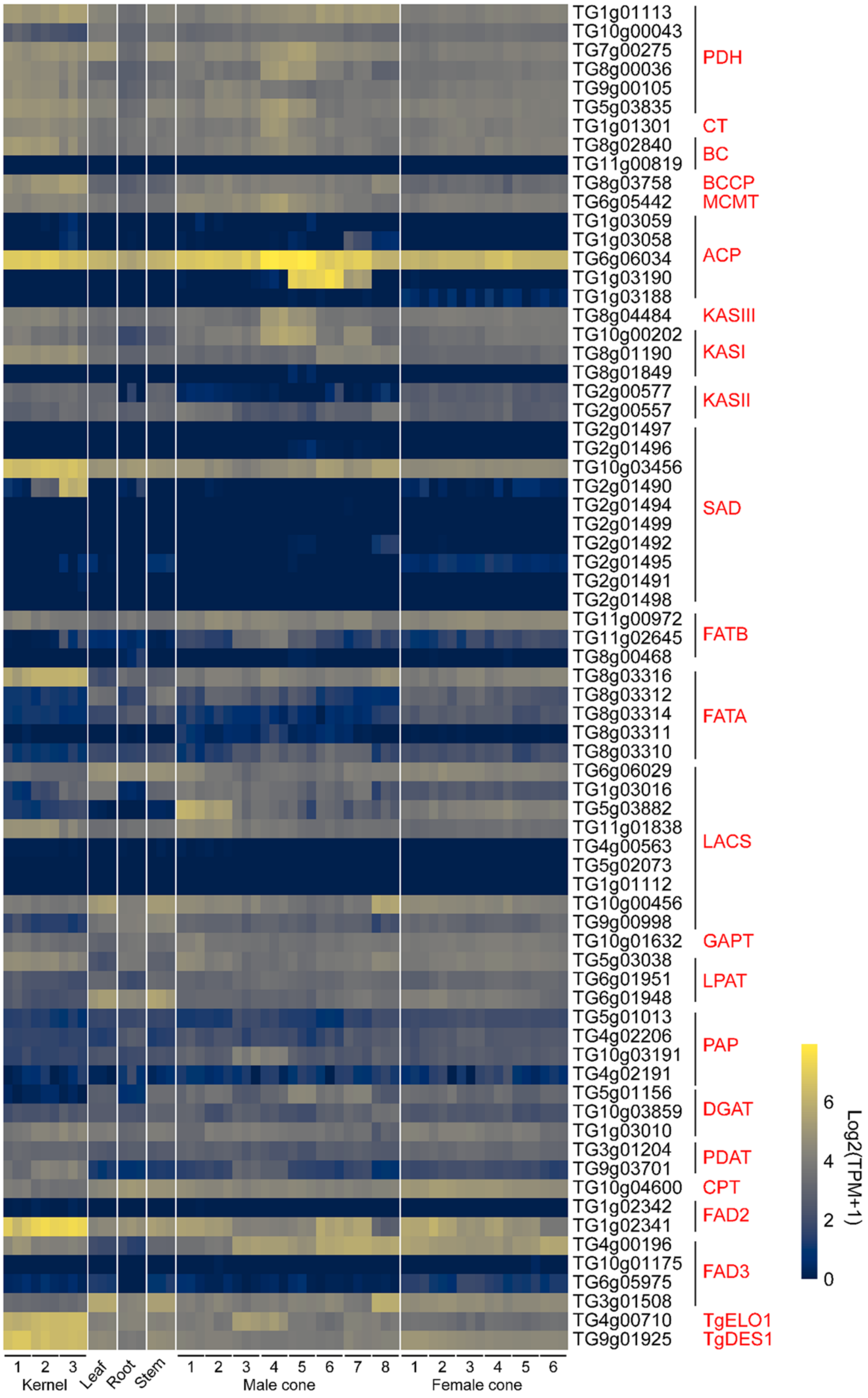

Supplementary Figure 12. Expression of fatty acid biosynthetic genes in different tissues of *T. grandis*.

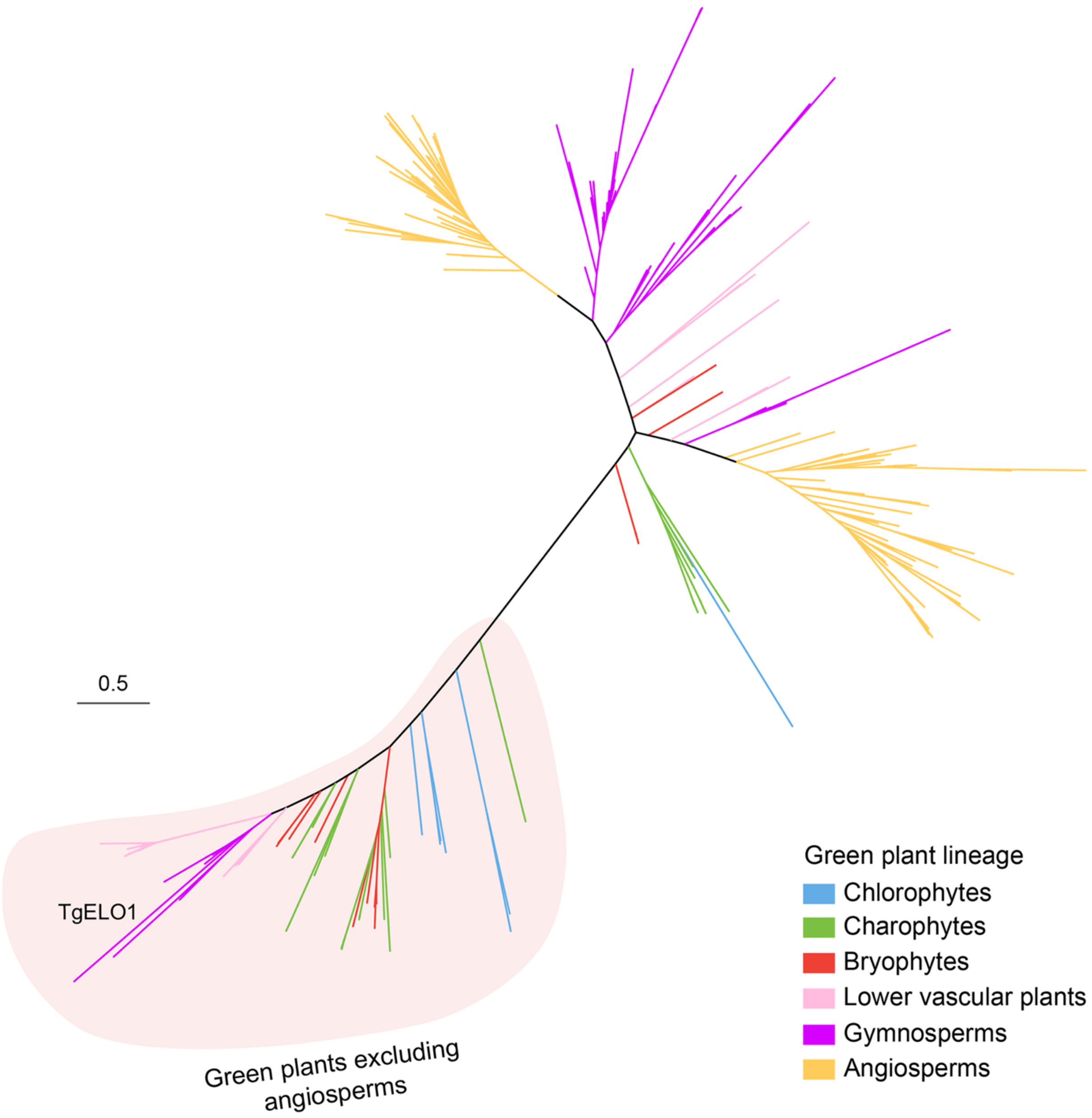

Supplementary Figure 13. Phylogeny of elongases. Protein sequences were aligned using MAFFT with linsi mode, and the phylogenetic tree was constructed using IQ-Tree with the best-fitting model (JTT+F+G4) and 1000 bootstrap replicates.

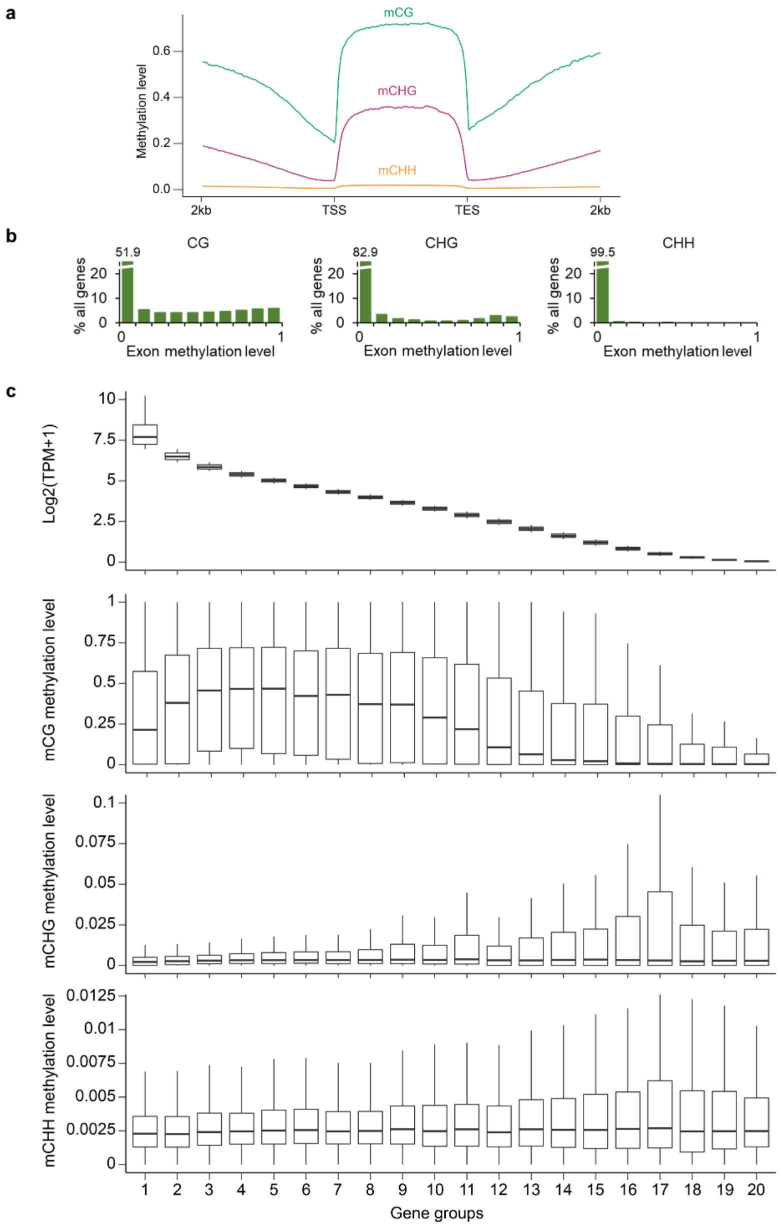

Supplementary Figure 14. DNA methylation of the seed genome. (**a**) Methylation levels in gene bodies and the 2-kb flanking regions. TSS, transcriptional start site. TES, transcriptional end site. (**b**) Methylation levels of exons in *T. grandis*. Genes were binned based on methylation levels and each bin represents a level of 0.1. (**c**) Comparison of gene expression and DNA methylation. Genes were categorized into 20 groups (N=1,519 for group 1-19 and N=1504 for group 20) based on the order of their expression levels. For each group, a boxplot was used to show the distribution of gene expression (top) and methylation levels at three different cytosine contexts (bottom). For the boxplot, the lower and upper bounds of the box indicate the first and third quartiles, respectively, and the center line indicates the median.

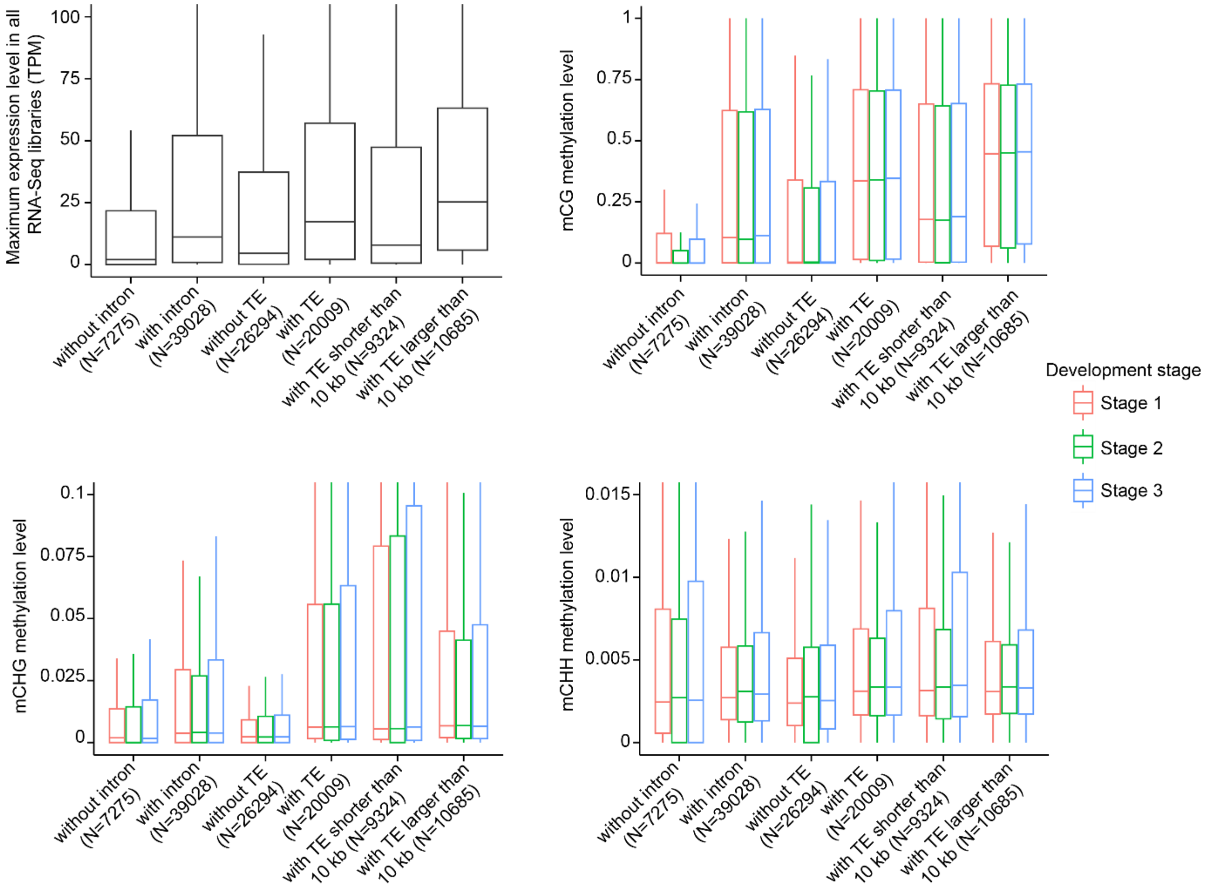

Supplementary Figure 15. Correspondence of expression and methylation of genes with or without TE insertions. Horizontal lines within boxes indicate the medium values. For each boxplot, the lower and upper bounds of the box indicate the first and third quartiles, respectively, and the center line indicates the median. N represents the number of genes used for each boxplot analysis.

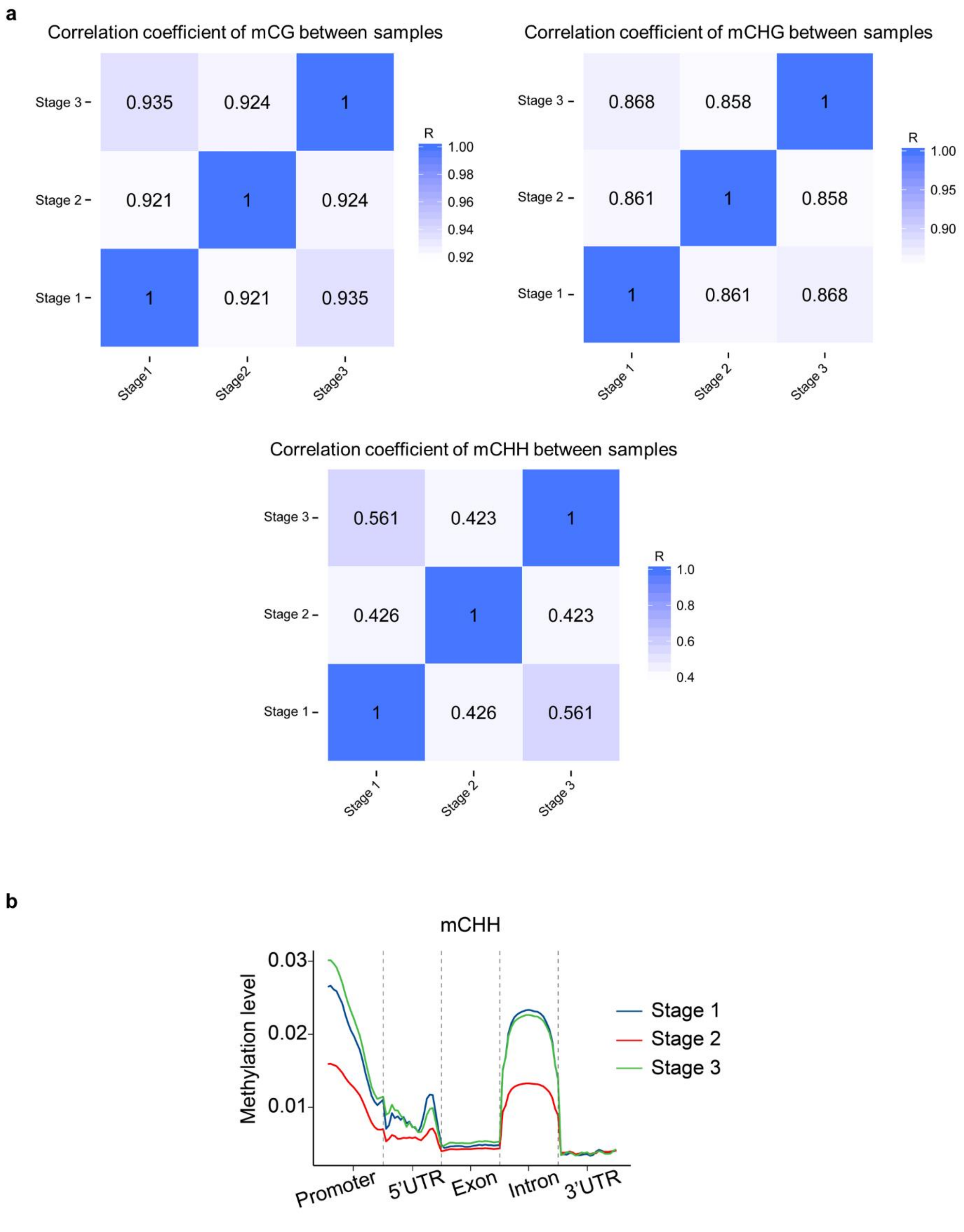

Supplementary Figure 16. DNA methylation at different stages of seed development. (**a**) Correlation coefficients of methylation profiles among different seed development stages. (**b**) mCHH methylation levels of different genomic features in seeds of *T. grandis*

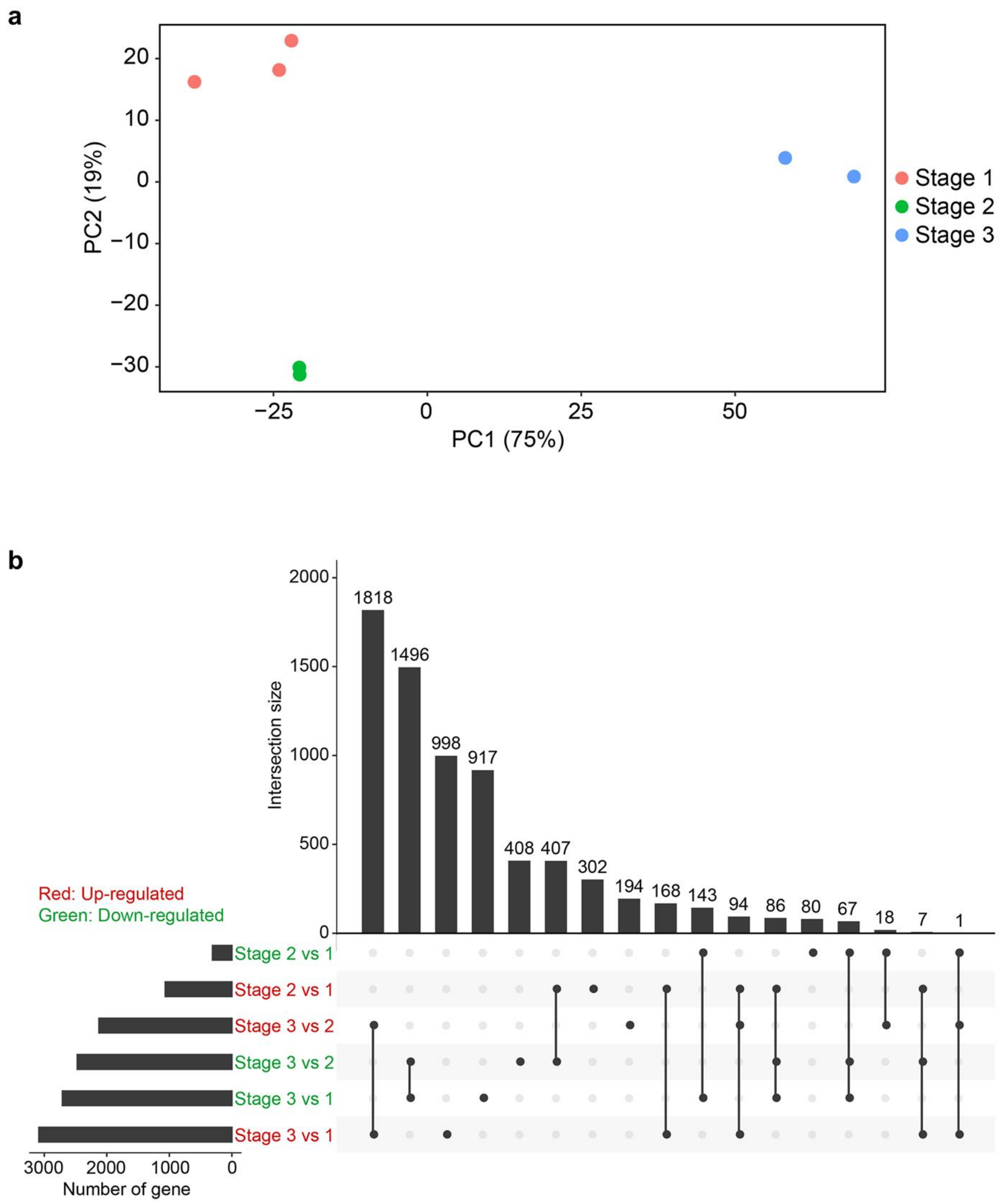

Supplementary Figure 17. Gene expression profiles at different seed development stages. (**a**) PCA plot of seed transcriptomes at three development stages. (**b**) Overlaps of differentially expressed genes among three seed development stages.

Supplementary Table 1. Statistics of the *T. grandis* genome assembly.

Estimated heterozygosity rate (%)	1.57
Estimated genome size (Gb)	20
Assembly size (bp)	19,050,820,213
Contig (#)	11,811
Contig N50 (bp)	2,822,555
Max contig length (bp)	16,786,683
Contig anchored (%)	99.13
Chromosome size (bp)	
Chr1	1,824,307,213
Chr2	1,166,843,293
Chr3	1,762,147,815
Chr4	1,717,786,663
Chr5	2,007,191,503
Chr6	1,957,849,926
Chr7	1,367,468,039
Chr8	1,668,700,658
Chr9	1,587,360,643
Chr10	2,000,250,390
Chr11	1,826,763,786
Repeat content (%)	59.8
Protein coding genes (#)	47,089
Functional annotation (homology evidence)	44,863
NR	39,320
Swiss-Prot	32,887
KEGG	30,564
InterPro	42,988
Pfam	30,393
GO	41,462
Supported by RNA-Seq reads	33,994
Supported by both RNA-Seq and protein homology	32,519
Supported by either RNA-Seq or protein homology	46,338

Supplementary Table 2. BUSCO evaluation of gymnosperm genome assemblies. BUSCO library used in this analysis was embryophyta_odb10. C: complete BUSCOs; S: complete and single-copy BUSCOs; D: complete and duplicated BUSCOs; F: fragmented BUSCOs; M: missing BUSCOs; n: total BUSCO groups searched.

Species	Genome size (Gb)	Number of filtered gene models	BUSCO evaluation	Mode of run
Torreya grandis	19.05	47,089	C:68.4%[S:61.0%,D:7.4%],F:17.4%,M:14.2%,n:1614	Genome
Torreya grandis	19.05	47,089	C:68.0%[S:60.8%,D:7.2%],F:17.2%,M:14.8%,n:1614	Protein
Ginkgo biloba	9.87	27,478	C:63.4%[S:58.0%,D:5.4%],F:19.1%,M:17.5%,n:1614	Protein
Gnetum montanum	4.07	27,491	C:83.8%[S:79.4%,D:4.4%],F:4.3%,M:11.9%,n:1614	Protein
Pinus tabuliformis	24.4	66,049	C:85.5%[S:70.9%,D:14.6%],F:7.1%,M:7.4%,n:1614	Protein
Sequoiadendron giganteum	8.13	41,631	C:50.0%[S:46.3%,D:3.7%],F:16.1%,M:33.9%,n:1614	Protein
Taxus wallichiana	10.9	44,007	C:84.9%[S:78.0%,D:6.9%],F:7.6%,M:7.5%,n:1614	Protein
Welwitschia mirabilis	6.86	25,593	C:75.4%[S:71.9%,D:3.5%],F:5.8%,M:18.8%,n:1614	Protein

Supplementary Table 3. Putative horizontally transferred genes in *T. grandis*.

ID	CAZyme calss	Possible origin	Function
TG8g01751	NA	Bacteria	Alcohol dehydrogenase
TG8g01760	NA	Bacteria	Alcohol dehydrogenase
TG8g02275	NA	Bacteria	Alcohol dehydrogenase
TG9g00555	NA	Bacteria	Beta-lactamase domain-containing protein
TG9g00557	NA	Bacteria	Beta-lactamase domain-containing protein
TG9g00559	NA	Bacteria	Beta-lactamase domain-containing protein
TG9g00560	NA	Bacteria	Beta-lactamase domain-containing protein
TG10g03948	NA	Bacteria	ATP-dependent DNA helicase
TG11g00029	NA	Bacteria	Phosphate acetyltransferase
TG2g01437	NA	Fungi	DNA-dependent protein kinase catalytic subunit
TG3g00899	NA	Bacteria	Light-independent protochlorophyllide reductase subunit N
TG3g00905	NA	Bacteria	Light-independent protochlorophyllide reductase subunit N
TG3g01731	NA	Bacteria	Light-independent protochlorophyllide reductase iron-sulfur ATP-binding protein
TG6g05734	NA	Fungi	UBC core domain-containing protein
TG5g03815	GH99	Bacteria	Endo-alpha-mannosidase
TG7g02803	GH99	Bacteria	Endo-alpha-mannosidase
TG7g02807	GH99	Bacteria	Endo-alpha-mannosidase
TG7g02808	GH99	Bacteria	Endo-alpha-mannosidase
TG7g02850	GH99	Bacteria	Endo-alpha-mannosidase
TG7g02851	GH99	Bacteria	Endo-alpha-mannosidase
TG7g02852	GH99	Bacteria	Endo-alpha-mannosidase
TG7g02855	GH99	Bacteria	Endo-alpha-mannosidase
TG7g02857	GH99	Bacteria	Endo-alpha-mannosidase
TG6g01556	GH71	Bacteria	Glycoside hydrolase family 71 protein
TG6g01527	GH71	Bacteria	Glycoside hydrolase family 71 protein
TG6g01533	GH71	Bacteria	Glycoside hydrolase family 71 protein
TG6g01532	GH71	Bacteria	Glycoside hydrolase family 71 protein
TG6g01534	GH71	Bacteria	Glycoside hydrolase family 71 protein
TG6g01555	GH71	Bacteria	Glycoside hydrolase family 71 protein
TG7g01923	GH71	Bacteria	Glycoside hydrolase family 71 protein
TG8g01578	GH103	Bacteria	Glycoside hydrolase family 103 protein
TG8g02665	CE4	Bacteria	Delta-lactam-biosynthetic de-N-acetylase
