# Supplementary material for: The Torreya grandis genome illuminates the origin and evolution of gymnosperm-specific sciadonic acid biosynthesis
Source: Nat Commun. 2023 Mar 10;14:1315. doi: 10.1038/s41467-023-37038-2 (PMC10006428; doi:10.1038/s41467-023-37038-2)
Supplement: Supplementary file 3 — Description of Additional Supplementary Files [file 41467_2023_37038_MOESM3_ESM.pdf]

Description of Additional Supplementary Files

Supplementary Data 1
Summary of DNA and RNA-Seq data used for genome assembly, gene prediction and transcriptome analysis.

Supplementary Data 2
Repeat sequences in the T. grandis genome assembly.

Supplementary Data 3
Homologues of genes involved in DNA methylation.

Supplementary Data 4
GO enrichment analysis of expanded gene families on the branch leading to the common ancestor of land plants, seed plants, angiosperms, or gymnosperms (excluding Gnetum and Welwitschia).

Supplementary Data 5
Selected gene families expanded in the T. grandis genome.

Supplementary Data 6
MIKC-type MADS-box genes in selected plant species.

Supplementary Data 7
Seed storage proteins in selected plant species.

Supplementary Data 8
CAZymes encoded by genome of selected plant species.

Supplementary Data 9
Statistics for whole genome bisulfite sequencing.

Supplementary Data 10
Transcription factors in genomes of selected plant species.

Supplementary Data 11
Differentially methylated regions (DMRs) between stage 1 and stage 2.

Supplementary Data 12
Differentially methylated regions (DMRs) between stage 1 and stage 3.

Supplementary Data 13
Differentially methylated regions (DMRs) between stage 2 and stage 3.

Supplementary Data 14

GO terms significantly enriched in DMR-associated genes in different comparisons of seed development stages.
