# Supplementary material for: The Torreya grandis genome illuminates the origin and evolution of gymnosperm-specific sciadonic acid biosynthesis
Source: Nat Commun. 2023 Mar 10;14:1315. doi: 10.1038/s41467-023-37038-2 (PMC10006428; doi:10.1038/s41467-023-37038-2)
Supplement: Supplementary file 18 — Reporting Summary [file 41467_2023_37038_MOESM18_ESM.pdf]

nature portfolio

Corresponding author(s): Zhangjun Fei, Xuepeng Sun, Jiasheng Wu

Last updated by author(s): Feb 24, 2023

Reporting Summary

Nature Portfolio wishes to improve the reproducibility of the work that we publish. This form provides structure for consistency and transparency in reporting. For further information on Nature Portfolio policies, see our Editorial Policies and the Editorial Policy Checklist.

Statistics

For all statistical analyses, confirm that the following items are present in the figure legend, table legend, main text, or Methods section.

n/a	Confirmed	
☐	☒	The exact sample size (*n*) for each experimental group/condition, given as a discrete number and unit of measurement
☐	☒	A statement on whether measurements were taken from distinct samples or whether the same sample was measured repeatedly
☐	☒	The statistical test(s) used AND whether they are one- or two-sided<br>*Only common tests should be described solely by name; describe more complex techniques in the Methods section.*
☒	☐	A description of all covariates tested
☐	☒	A description of any assumptions or corrections, such as tests of normality and adjustment for multiple comparisons
☐	☒	A full description of the statistical parameters including central tendency (e.g. means) or other basic estimates (e.g. regression coefficient) AND variation (e.g. standard deviation) or associated estimates of uncertainty (e.g. confidence intervals)
☐	☒	For null hypothesis testing, the test statistic (e.g. *F*, *t*, *r*) with confidence intervals, effect sizes, degrees of freedom and *P* value noted<br>*Give P values as exact values whenever suitable.*
☒	☐	For Bayesian analysis, information on the choice of priors and Markov chain Monte Carlo settings
☒	☐	For hierarchical and complex designs, identification of the appropriate level for tests and full reporting of outcomes
☒	☐	Estimates of effect sizes (e.g. Cohen's *d*, Pearson's *r*), indicating how they were calculated

Our web collection on statistics for biologists contains articles on many of the points above.

Software and code

Policy information about availability of computer code

Data collection	No software was used in data collection.
Data analysis	We used freely available bioinformatics softwares, including: hifiasm (version 0.8-dirty-r280), Purge Haplotigs (version 1.1.0), Trimmomatic (version 0.36), ALLHiC (version 0.9.8), BWA-MEM (version 0.7.17-r1188), LTR_Finder (version 1.2), RepeatModeler (version 1.0.3), RepeatMasker (version 4.1.2-p1), LTR-harvest (version 1.6.1), MUSCLE (version 3.8.1551), AUGUSTUS (version 3.4.0), GlimmerHMM (version 3.0.4), SNAP (version 2006-07-28), geneid (version 1.2), GENSCAN (version 1.0), tblastn (version 2.9.0+), GenBlastA (version 1.04), GeneWise (v.2.4.1), TopHat (version 2.1.1), Cufflinks (v.2.2.1), EVidenceModeler (version 1.1.1), BUSCO (version 5.1.3), InterProScan (version 5.51-85.0), OrthoFinder (version 2.5.4), IQ-TREE (version 2.1.3), CAFE (version 4.2.1), MCScanX (versrion 2015), PAML (version 4.8a), Bowtie (version 1.2.3), Bismark (version 0.16.3), DSS (version 2.38.0), STAR (version 2.7.10a), HTSeq-count (version 0.6.1p1), DESeq2 (version 1.31.2)

For manuscripts utilizing custom algorithms or software that are central to the research but not yet described in published literature, software must be made available to editors and reviewers. We strongly encourage code deposition in a community repository (e.g. GitHub). See the Nature Portfolio guidelines for submitting code & software for further information.

March 2021

Data

Policy information about availability of data

All manuscripts must include a data availability statement. This statement should provide the following information, where applicable:

- Accession codes, unique identifiers, or web links for publicly available datasets
- A description of any restrictions on data availability
- For clinical datasets or third party data, please ensure that the statement adheres to our policy

KEGG (https://www.genome.jp/kegg/);SwissProt and TrEMBL (https://www.uniprot.org/); GO (http://geneontology.org/); InterPro (https://www.ebi.ac.uk/interpro/); Genome assembly and raw reads for genome, transcriptome and methylome sequencing have been deposited in the CNGB Sequence Archive (CNSA) of China National GeneBank DataBase (CNGBdb, https://db.cngb.org) with the accession number CNP0003453. Genome assembly and annotation are also accessible through https://figshare.com/articles/dataset/Genome_assembly_and_annotation_of_Torreya_grandis/21089869

Human research participants

Policy information about studies involving human research participants and Sex and Gender in Research.

Reporting on sex and gender	NA
Population characteristics	NA
Recruitment	NA
Ethics oversight	NA

Note that full information on the approval of the study protocol must also be provided in the manuscript.

Field-specific reporting

Please select the one below that is the best fit for your research. If you are not sure, read the appropriate sections before making your selection.

☒ Life sciences ☐ Behavioural & social sciences ☐ Ecological, evolutionary & environmental sciences

For a reference copy of the document with all sections, see nature.com/documents/nr-reporting-summary-flat.pdf

Life sciences study design

All studies must disclose on these points even when the disclosure is negative.

Sample size	One plant of Torreya grandis was selected for genome sequencing and assembly, and the same plant was used for transcriptome sequencing of different tissues for genome annotation. Differential gene expression analysis was performed in three individual plants as independent biological replicates. Each plant was used for small RNA sequencing and methylome analysis.
Data exclusions	No data exclusions in this study
Replication	Three biological replicates were used for transcriptome sequencing of gene expression studies. All attempts at replication were successful. One plant (or replicate) was used from genome, sRNA, methylome sequencing, and all attempts were successful. qPCR and measurements of SCA content were performed with three biological replicates and all attempts at replication were successful.
Randomization	Biological replicates for transcriptome sequencing of gene expression studies and measurements of SCA content were randomly selected.
Blinding	Not applicable. The authors collected samples, conducted the experiments and performed all data analyses so blinding is not possible in this study

Reporting for specific materials, systems and methods

We require information from authors about some types of materials, experimental systems and methods used in many studies. Here, indicate whether each material, system or method listed is relevant to your study. If you are not sure if a list item applies to your research, read the appropriate section before selecting a response.

Materials & experimental systems

n/a	Involved in the study
☒	☐ Antibodies
☒	☐ Eukaryotic cell lines
☒	☐ Palaeontology and archaeology
☒	☐ Animals and other organisms
☒	☐ Clinical data
☒	☐ Dual use research of concern

Methods

n/a	Involved in the study
☒	☐ ChIP-seq
☒	☐ Flow cytometry
☒	☐ MRI-based neuroimaging
